# Percutaneous septal ablation for left mid-ventricular obstructive hypertrophic cardiomyopathy: a case report

**DOI:** 10.1186/1471-2261-6-15

**Published:** 2006-04-10

**Authors:** Istemihan Tengiz, Ertugrul Ercan, Emin Alioglu, Ugur O Turk

**Affiliations:** 1 Central Hospital, Cardiology Department, 1644 sk. No:2/2, Bayrakli, Izmir, Turkey

## Abstract

**Background:**

Mid-ventricular obstructive hypertrophic cardiomyopathy (MVOHC) is a rare type of cardiomyopathy. The diagnosis is based on the hourglass appearance on the left ventriculogram and the presence of pressure gradient between apical and basal chamber of the ventriculum on the hemodynamic assessment.

**Case presentation:**

The present case represents successful percutaneous treatment with septal ablation to patient with MVOHC associated with systolic anterior motion of the mitral valve and obstruction at both the mid-ventricular and outflow levels.

**Conclusion:**

Alcohol septal ablation has been proposed as less invasive alternatives to surgery in patients with MVOHC.

## Background

Hypertrophic cardiomyopathy (HC) is a hereditary myocardial disorder, caused by mutations of sarcomeric proteins [1]. In roughly 25% of cases of HC there is associated obstruction to left ventricular (LV) outflow [1]. Obstruction can occur at several locations within the ventricle, depending on the distribution of hypertrophy, including a) the mitral valve level in association with systolic anterior motion (SAM) of the mitral valve; b) the mid-ventricle; or c) within the cardiac apex [1].

In about 5% of patients a mid-ventricular obstruction (MVO) can be observed [2]. These patients are often symptomatic from hemodynamic causes and are also prone to symptomatic and even lethal ventricular arrhythmias. MVO may be associated with hypertrophy of the papillary muscle(s) and apical LV aneurysm. MVO usually presents as local obstruction but can be associated with SAM and obstruction at both the mid-cavitary and outflow levels [1].

During the past 10 years, atrio-ventricular sequential pacing and alcohol septal ablation have been proposed as less invasive alternatives to surgery for patients who fail to respond to pharmacologic therapy [3,4]. However, atrio-ventricular sequential pacing is not currently thought to be dramatically beneficial [5,6]. We report on percutaneous septal ablation in a patient with left mid-ventricular obstructive hypertrophic cardiomyopathy (MVOHC) associated with SAM of the mitral valve and obstruction at both the mid-ventricular and outflow levels, which seems to be an alternative therapy in these patients.

## Case presentation

**Figure 1:**
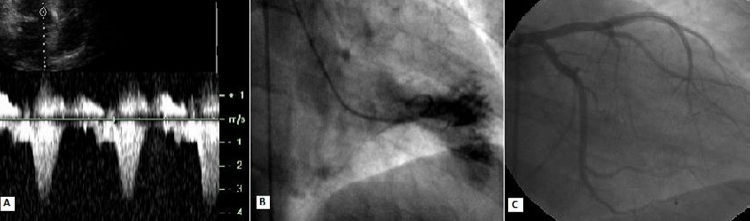
A: Pre-procedural continuous-wave Doppler recordings, B: Hyperkinetic left ventricle, C: Dominant first septal branch.

**Figure 2:**
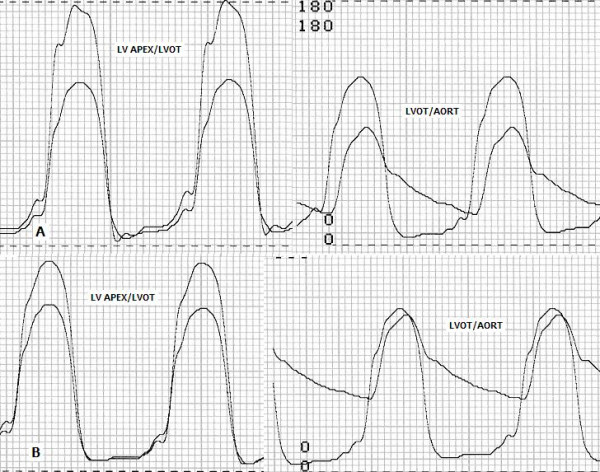
A: Pre-procedural invasive pressure recordings, B: Post-procedural invasive pressure recordings.

A 34-year-old man with HC (primarily diagnosed 6 years ago) was referred to our clinic with the complaints of palpitation, chest pain and dyspnea on exertion, despite medication with 200 mg metoprolol daily. His functional capacity was New York Heart Association (NYHA) class III. He had no family history of HC. The fourth heart sound and moderate systolic murmur in mitral area radiating to mid-clavicular line were heard at cardiac auscultation. On electrocardiogram, sinus rhythm, left axis deviation and repolarization abnormalities (negative T vawes) were seen. Chest X-ray showed neither cardiomegaly nor pulmonary congestion. Echocardiographic examination was done to assess the LV anatomy and revealed normal dimensions of the LV at end-diastole (41 mm) and end-systole (19 mm), SAM of the mitral valve with moderate mitral regurgitation, left atrial dilatation (55 mm) and hypertrophic interventricular septum (mid:25 mm, basal:27 mm). LV ejection fraction was 75%. Continuous-wave Doppler measurements showed a peak systolic resting gradient of 46 mmHg, with an increase to 96 mmHg at Valsalva's maneouvre at the LV outflow (Figure 1A). Coronary angiography and invasive hemodynamic assessment were done. Coronary angiograms showed no narrowing of major epicardial coronary arteries. The left ventriculography showed a hyperkinetic contraction pattern at the mid-ventricle with a narrow muscular tunnel between the LV apical and bazal cavities (Figure 1B). Hemodynamic assessment revealed a significant intra-ventricular pressure gradient of 56 mmHg (LV apex 178/10 mmHg, LV outflow 122/12 mmHg) with mild LV outflow tract pressure gradient of 30 mmHg (aorta 92/64) (Figure 2A). We finally diagnosed MVOHC associated with SAM of the mitral valve and obstruction at both the mid-ventricular and outflow levels.

**Figure 3:**
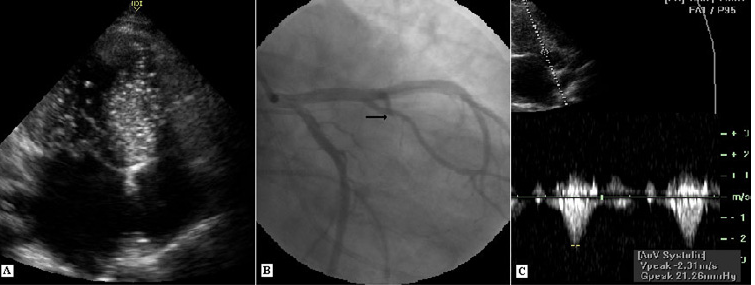
A: The echocontrast appearance in hypertrophic muscle segment, B: No angiographic appearance after alcohol injection in first septal branch (Black arrow), C: Post-procedural continuous-wave Doppler recordings.

**Figure 4:**
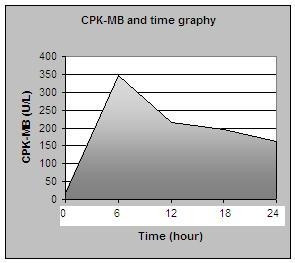
The size of the caused infarction estimated by areas under the CPK-MB versus time graph's curve.

The patient was informed about the therapeutic options and gave her written consent to percutaneous septal myocardial ablation. The first septal branch was dominant (diameter: 2.1 mm) and showed prominent septal myocardial distribution (Figure 1C). The distance between the left anterior descending coronary artery ostium and the origin of the first septal branch was 24.4 mm. Other distal septal branches were very small. It was considered that the first septal branch ablation may be effective in decreasing the intraventricular pressure gradient. The procedure was performed as described elsewhere [7]. The floppy wire (Choice scimed guide wire, 0.014", 300 cm, Boston Scientific, Miami, USA) was crossed to first septal branch which estimated target vessel. A 2.5 × 20 mm over the wire balloon (Maverick OTW PTCA dilatation catheter, Boston Scientific, Maple Grove, USA) was placed in the proximal part of the vessel. The echo contrast agent (Echovist, Schering) was administered selectively in the estimated vessel under simultaneous color Doppler echocardiography. Prior to alcohol injection, balloon position was verified by myocardial contrast echocardiography (Figure 3A). When the balloon was inflated and transient occlusion constituted, hemodynamic measurements were repeated. After that demonstrating the significant reduction in intra-ventricular pressure gradient, the vessel was occluded by injection of 3 cc absolute alcohol in portions of 1 cc/min. (Figure 3B). Repeated hemodynamic measurements found a decrease of the post-extrasystolic gradient to 18 mmHg at the mid-ventricular level with no resting gradient in the LV outflow level (Figure 2B). Patient had slight chest pain during the procedure. Complications such as conduction abnormality were not seen. The post-procedural electrocardiogram showed ST segment elevation (maximal 6 mm) in V_1–2 _with reciprocal ST segment depression in II, III and aVF. Maximal creatine kinase rise was 1591 U/l with a MB fraction of 347 U/l. The size of the caused infarction was estimated by areas under the CPK-MB versus time graph's curve. SAS 9.1 statistical software was used to calculate area under the curve. Area under the curve was 5070 U/L hour for CPK-MB (Figure 4).

The post-procedural echocardiographic examination was done before the discharge and showed reductions of left atrial dimension to 46 mm and interventricular septum thickness to 20 mm with septal hypokinesia and mild mitral regurgitation without SAM of the mitral valve. LV end-diastolic and end-systolic diameters were 49 mm, 32 mm respectively. Continuous-wave Doppler measurements showed 21 mmHg a peak systolic resting gradient, with an increase to 36 mmHg at Valsalva's maneouvre (Figure 3C). The patient was discharged after an uneventful hospital stay of 5 days. Medication with 100 mg metoprolol was continued at discharge.

**Figure 5:**
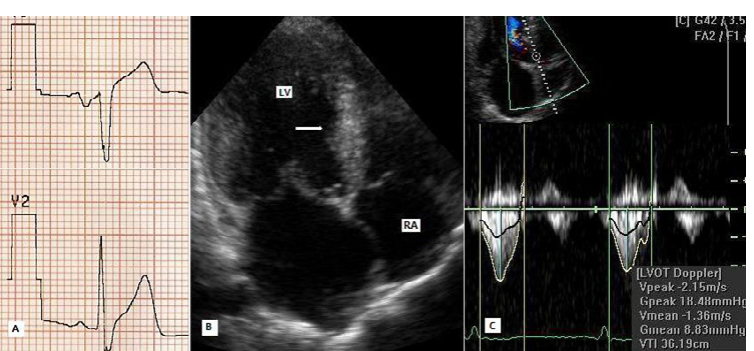
A: Post procedural ECG, B: Post-procedural apical 4 chamber view after six months, C: Continuous-wave Doppler recordings after six months.

At 6 months' follow-up, the patient was asymptomatic and her functional capacity was improved (NYHA class I). Physical examination showed mild systolic murmur in mitral area. The electrocardiogram showed sinus rhythm, left axis and no Q wave in V_1–2 _(Figure 5A). The control echocardiographic examination showed reduction of interventricular septum thickness (mid:18 mm, basal:16 mm, Figure 5B) and mild mitral regurgitation without SAM of the mitral valve. LV ejection fraction was 67%. Continuous-wave Doppler measurements showed 18 mmHg peak systolic resting gradient, with an increase to 31 mmHg at Valsalva's maneouvre (Figure 5C).

## Conclusion

Obstruction at lower levels of the ventricle simply reflects partial obliteration of the ventricular cavity by the contracting hypertrophied muscle in the face of continued ejection of blood. An asynergic or aneurismal apical chamber in the absence of fixed coronary artery disease has been previously described in patients with MVO [8]. It has been suggested that the development of apical aneurysm may be secondary to increased afterload and high apical pressure resulting from a systolic MVO [9]. Therefore, relief of the high apical pressure may be necessary preventing further asynergy.

Myectomy has been employed primarily in patients with obstruction at the mitral valve level. Because obstruction primarily relates to SAM of the mitral valve, some have recommended mitral valve replacement as the most direct method for relieving obstruction [1]. Mitral valve replacement has also been recommended for patients with direct insertion of the papillary muscle into the anterior mitral leaflet and associated MVO, although this strategy is undesirable for young patients. In these cases, extending and broadening the myectomy in the mid-ventricle and mobilizing the papillary muscle with valve preservation has been reported with good success [1].

Non-surgical treatment options for the obstructive cardiomyopathies are performed to increasing number of patients to reduce hospitalization duration and cost. With DDD pacing the outflow gradient can be reduced more than 30%. Randomized trials have shown a limited hemodynamic and clinical improvement in severely symptomatic patients with hypertrophic obstructive cardiomyopathy [10]. Although there are a few report of a therapeutic effect of DDD pacing in MVO [11,12], it can not be recommended for routine application due to failure of reliable predictability of individual therapeutic success [5].

Nonrandomized reports comparing septal ablation with myectomy describe similar degrees of LV outflow tract gradient reduction with follow-up between 3 months and 1 year [13]. Additionally, improvements in clinical endpoints, such as quality of life and functional status, have been comparable, suggesting that septal ablation is a safe and effective alternative to myectomy in select patients. However, occlusion of the septal branches is generally ineffective for reducing the intra-ventricular pressure gradient if they do not supply the obstructive muscle mass. The most important problem is the identification of the target vessel. Variability in the size and distribution of the first septal branch in patients with HC is substantial. Areas of the heart other than the basal septum are supplied in some patients by the first septal branch. In other patients the first septal branch is not supply the entire basal septum. A detailed evaluation of the distribution of the first septal branch may be necessary in all patients with HC who are undergoing alcohol septal ablation [14]. Echocardiographic monitoring is indispensable in order to identify the target vessel and to exclude alcohol misplacements.

The fourth septal branch ablation for pressure gradient reduction in patients with MVOHC has been reported by Seggewiss et al [15]. The present case had MVOHC associated with SAM of the mitral valve and obstruction at both the mid-ventricular and outflow levels. In addition, the first septal branch was pretty dominant and considered that alcohol ablation can be effective in decreasing the intra-ventricular pressure gradient. Likewise, the echocontrast injection in the first septal branch resulted with the largest echocontrast view in hypertrophic muscle segment. Furthermore, transient occlusion of the vessel by balloon inflation resulted in significant decrease in intra-ventricular pressure gradient. Therefore, the first septal branch was assumed as a target vessel and occlusion of the vessel resulted with a significant and sustained reduction of symptoms.

There are multiple factors cause severe arrhythmias during the procedure, including ischemia, infarction, and the presence of an arrhythmogenic myofibrillar substrate, local-tissue toxicity from the alcohol and echocontrast agent. Although were not seen in our case, total AV block and ventricular fibrillation may occur with application of echovist in the systemic arterial circulation. Therefore, levovist is appropriate echo contrast agent for septal ablation [16]. Development of LV myocardial damage, heart failure, ventricular septal defect, dysfunction of papillary muscles and severe rhythm disturbances such as permanent or transient atrioventricular block required pacemaker implantation are important limitations of this procedure [17].

In summary, percutaneous septal ablation may be useful in patients with MVOHC accompanying with LV outflow tract obstruction. Echocardiographic monitoring in alcohol induced percutaneous myocardial ablation enables the widening of morphological indication to patients with MVOHC. Septal ablation in MVOHC is not classical and wide accepted treatment in contrast to the subaortic type LV obstruction. Possible complications and unknown long-term effects require careful patient selection as well as experience in interventional cardiology and hypertrophic cardiomyopathy.

## Competing interests

The author(s) declare that they have no competing interests.

## Authors' contributions

IT, EE drafting the article, performed percutaneous intervention, critical revision of the article, and final approval of the version.

EA, UOT echocardiographic examinations.

All authors read and approved the final manuscript.

## Pre-publication history

The pre-publication history for this paper can be accessed here:


